# Activating Embodied Imagination During COVID-19: A Performative Reflexive Autoethnography

**DOI:** 10.1177/1077800420962474

**Published:** 2021-09

**Authors:** Mark B. DeGarmo

**Affiliations:** 1 Mark DeGarmo Dance (AKA Dynamic Forms, Inc.), New York, NY, USA

**Keywords:** bricolage, COVID-19, embodied imagination, performative reflexive autoethnography

## Abstract

Embodied imagination is a learning theory that reverses the accepted Western “think first, then act” learning sequence though movement improvisation followed by reflection and reflective methods across verbal and nonverbal, including embodied-kinesthetic, modalities. Healing the Cartesian divide might have positive effects on world cultures and people across socioeconomic strata, especially urgent during the COVID-19 pandemic as multiple disruptions to daily life have quickly increased uncertainty and stress, compromising health and well-being, especially of traditionally marginalized excluded People of Color. Expanding the performative reflexive autoethnographic project through embodied imagination broadens and deepens this global, transcultural, transdisciplinary effort through the human body, traditionally not considered human thinking’s locus. Benefits across global societies include greater self-care, the ability to act effectively quickly in response to a world with exponentially increasing complexity, and awareness that creativity is a global communitarian human birthright, not a rarity relegated to exceptional people.

**Table:** 

Global Crises	Every day I’m faced
Overlapping global crises confront the United States. The COVID-19 pandemic might characterize ours as the “Century of Pandemic,” as the 20th earned “Century of Genocide (Kapuściński, 2001). Concurrently, Black Lives Matter political protests emerged in outrage across the United States and worldwide over the videotaped murder of George Floyd by strangulation and suffocation, over 8 min, 49 s, by a Minneapolis law enforcement officer and trainer with three others participating or passively witnessing on May 25. Additionally, in an election year, the United States’s national political polarization reveals deep, prolonged “cultural wars” informed by race plus broad and deep internalized-systemic racism (Jones, 2000). Mounting critiques across U.S. society include those of military leaders. White supremacy surfaces as the United States’s founding principle that continues segregating by race first, class second. Across U.S. educational systems, the histories of Blacks, Peoples of Color, and American Indian and Global Indigenous Peoples are taught inadequately if at all (DeGarmo, in press; Jiménez, 2016; United Nations, 1948, 2007).	Every day I’m faced with my latest daily failures. I feel like a fifth grader special needs boy held back in third grade with total dyslexia-techno-phobia unable to read the screen, perceive the meaning of the computer icons, unable to translate from the new, improved visual world to my body, my embodied-analog- way-of-knowing, understanding, doing, and sense-making from my senses, not Bill Gates’ genius, from my stupidity. I wake to feel myself a total failure for 10 weeks now in virtual lock-down hell. Encased in amber, a relic from the recent analog-past and born in the 20th century. I am a child left-behind with no chance for mourning, no gatherings, not a single touch. Virtual glances, virtual distancing. I feel lost, blinded, blindsided by today’s world. (DeGarmo, 2020a)
Therefore, awareness is growing, albeit slowly and without consensus, among the White geographically dispersed general public that U.S. genocide and slavery built the country’s and possibly their own wealth, and never ended with treaties and abolition. These massive legacies continue to inform, pollute, and undermine U.S. social, educational, political, economic, housing, healthcare, and police funding and enforcement policies and practices. Meanwhile, during the past 5 months, March to July 2020, on the microscopic level, there quickly emerged an urgent need for self-care and self-protection in national, regional, and local environments displaying rapidly increasing polarization and disruption of public and communitarian discourses. A newly charged public posturing materialized, grounded in confrontation rather than expression of alternative views with hopes of creating synthesis and new shared understandings of complexity and complex issues. Increased stress levels across multiple complex toxic factors impacted ways of daily living and doing business, resulting in increased mental illness, domestic violence, and self-inflicted harm. Many students and their families, unable to meet the technical knowledge, equipment, and broadband internet access requirements, did not show up for online learning mandated when schools were abruptly closed. A colleague shared, however, that even MIT and Harvard were unequipped to pivot quickly to online teaching and learning.	Global Disaster . . . (and i showed up) Global disaster. . . and i showed up. For beginning-middle-or-end? My embodiment is existentially threatened. Theory appears, but practice calls me. i peer through a visual peephole outwardly and feel through a kinesthetic portal inwardly
**Reflections on Aging, Death, and Dying** I am a dancer, choreographer, writer, and researcher, and I launched my dream to “become” a dancer and choreographer as a 15-year-old about 50 years ago. I have been reflecting on aging, death, and dying during COVID-19’s sheltering-in-place mandates. Embodied imagination, movement improvisation followed by reflection and reflective practice across various verbal and nonverbal modalities, reverses the accepted Western understanding of knowledge construction (DeGarmo, 2007). Embodied imagination as a learning theory posits that action followed by reflection is the sequence for accessing embodied imagination.	toward embodied cognition’s freeze—fight—flight responses stymied, gunked up by COVID. By human hands, human breath, human contact left behind like shit stains on a Lower East Side street.
Through the process of participating in the “massive and microscopic sensemaking” challenge posed by Markham and Harris (2020) via my performative reflexive autoethnography, I have learned that embodied imagination’s implied cycling action always begins again in life. As one ages, however, in the sense of having more experiences to draw upon and stories to tell, particular ways of knowing or working through a topic reflexively might emerge. With aging, embodied imagination is not a tight circling or spiraling. It is a flow of movement whether through air or water with much broader loops, more akin to submerging and surfacing in the water that create a pattern more like an eddy or reverberations of ripples. It is a dance with various incarnations of oneself.	An invisible microscopic link to our extinction. The White Gods have returned, again, as they did, as prophesied. Embodied imagination: movement-improvisation- and-reflection feel to be my saving grace, my core, self-care during a first global tsunami, humankind’s warning from Mother Earth to her children.
My intersectional identities include founding and running a New York City not-for-profit dance performance organization and developing dance education programs for public schools since the 1980s (Corcoran, 2018). Schools in the United States, starting at the elementary level, teach the accepted U.S. method of “think first,” then possibly act, and if so, act judiciously, cautiously, and sometimes apologetically. Concurrent with perpetuating Western cultural norms inherited from the positing of the Cartesian divide, this accepted learning and teaching methodology perpetuates practical and negative everyday consequences. What has reflexively surfaced for me is realizing that I am still investigating whether my personal Cartesian divide is healed, or even healing, through my embodied imagination. I have, however, come to recognize that bricolage takes place over time and through stories, as well as, through the complexity of my own embodied imagination learning practice.	My experiential inquiry has well begun, if not begun well. Survival seems to suggest: “Best to keep moving. Movement is life itself.” Hence, questions arise as words: “Will i ever embracemy beloved again? Will my loved ones ever hold me?
**Bricolage and Performative Reflexive Autoethnography** Bricoleurs are obsessed with recovering meanings about the physical, social, political, psychological, and educational worlds that have been lost, that have fallen through the disciplinary cracks of modernism. **Such losses are** **especially severe in the domains of** **the ecological, the emotional, the** **unconscious, the ideological, and** **the cultural, as many research** **orientations are simply not** **prepared to produce knowledge** **within these areas** [Emphasis added]. (Kincheloe, 2004, p. 89)	Will the body politic hold? Will i? Will my nonprofit dance company and
Disruption of previous patterns of cognition and habitual ways of moving inherited from cultural, educational, familial, and personal experiences, assumptions, and normativity is often an aspiration for the performative improvisational composer and embodied bricoleur, regardless of the degree to which originality or innovation for the individual is possible. Embodied imagination offers a methodology to be further developed as part of the expanded bricolage project. It disrupts through self-directed and somatic (i.e., body-based) inquiry and performative research methods, cultural normativity through juxtaposition, multiple perspective-taking, and layering and removing layers. As with other forms of bricolage, one rationale for its research purpose is to uncover or bring to the surface deeper levels of implicit and tacit material through patterns otherwise unperceived, unseen, and unfelt via the kinesthetic and proprioceptive senses. The embodied methodological research bricoleur’s attention to “rigour and complexity” includes use of an armature to prepare for activating embodied imagination within an improvisational time-space-energy-relationship continuum in a performative moment. The Chinese calligrapher’s single-stroke moment of insight grounded in lifelong study and practice is a metaphor for an armature’s purpose. My bricolage armature during COVID-19 included throwing open my New York City apartment’s windows, as my mother always did mid-winter, March 13–17 while trying to mobilize in exodus to my country home 2 hr north; looping the same hike on my road; repeating words and phrases; and swimming to “bypass cognitive” domination and logocentric decision making (Cedillos, 2012).	our 9 teammates, for whom i am responsible? Will i return to The City or stay put in Mother’s birthplace cradle for me straddling the Catskills and Taconic Mountains? Archetypal forces are aligning for what is next, what we witness, how we live to perform the tale, its poem, and dance the materials at hand.
The complexity that the embodied bricoleur acknowledges and encounters through rigor includes the otherwise invisible yet embedded crystalline structures that refract and reflect the multiple dimensions and levels of the bricoleur’s past experiences, emotions, trainings, explorations, injuries, pleasures, and physiological challenges. These crystalline structures are embedded landscapes of the human body’s record of one person’s life’s journey. It is my reflexive “coming to know” over a life span and through remembering.	Are we at the edge of a black abyss? Is this, or worse,how it ends?
My embodied bricolage and embodied imagination practices and methods include: preparing, entering, gathering, performing, exiting, and reflecting. One prepares the performative space, enters it with detachment and expectation; gathers the forces one needs, performs the task at hand with the tools required to do so, exits the performance space, returning to pre-performance physical levels, and reflects on the meaning of the cycle. Performative reflexive autoethnography, as inquiry approach and theory are new to me (Alexander, 2005; Bialostozky Judisman, 2016; Holman Jones, 2005; Markham, 2005; Soyini Madison & Hamera, 2006; Spry, 2001). Yet I know them well as a practicing creative artist and performer. Constructivist grounded theory provided the emergence from practice to theory of embodied imagination. Through this massive and microscopic process, I have learned that the personal stance can inform understanding of the massive forces emerging during COVID-19. I have experienced a tremendous amount of emotional and physical pain that surfaced through my body over the past 5 months. I realized that the themes of aging, death, and dying while not unknown to a man who has faced a lifetime of trauma have looped back into my sphere of meaning-making through this performative reflexive autoethnography. **Benefits of, and Learning Through, Embodied Imagination During COVID-19** Embodied imagination can play a key role in self-directed learning and healing and in a deeper understanding and appreciation of self and others, including the Earth. Hopefully, there are ways through (the anxiety, uncertainty, unknowable) by focusing on our bodies and self-care (acknowledging this situatedness is privileged, not universal; yet there are benefits across socioeconomic strata). There are teaching, learning, healing, growing/expanding capacity benefits. Some findings are a greater ability to trust the process, be present, find balance by oneself in relative isolation, and ground through the body first (self), to reach out to others, and the larger world (social media, tech), and the Earth.	Where are the beginnings? Who am i?” Paradoxically inklings surface to consciousness that my life’s repeated history of physical and emotional traumas now serves me, strengthens me, reflects the wisdom of the elders, the storytellers, and the dancing fools. My ancestors’ presence through my flesh linking molecularly to the stars themselves guides me. Micro and macro merging in my bones, flesh, organs, sinews, connective tissues channel performativity. We are one human organism on our planet and embody violence and love. i **feel** darkness. *i* **see** light. These are my ideas. (DeGarmo, 2020b)
Benefits of Activated Embodied Imagination During COVID-19: • Responding rapidly and effectively to quickly changing volatile environments and high degrees of uncertainty; • Maintaining physical, emotional, social, and psychological well-being during physical isolation, solitude, and distancing; • Achieving greater comfort and confidence exploring and experimenting with new technologies, forms, and ways of interfacing with self, others, and the environment, including performative reflexive autoethnography; • Connecting the internal, micro-environmental leadings of direct human experience with experiences of the global digital environment; and • Practicing transcultural transdisciplinary problem-solving across multiple levels and dimensions, including issues involving humans, machines, and the planet. My experience knowing embodied imagination’s learning sequence supported navigating uncertainties thus far. I found I had to “restart/reboot” (as much as I mistrust using this ICT metaphor) my body-mind-spirit-emotions. I am finding my way with greater reassurance only recently, as I started writing this paper, that is, shedding my anxiety and consciously wanting to release, not hold, it. **Keeping on During COVID-19** Our messy, often segregated and compartmentalized, lived lives just got a whole lot messier in the United States during COVID-19. As I do in my life’s, artistic, and research generative, analytical, and interpretive processes, I created masses of audiotaped, photo/videographed, visual arts, and written materials functioning as performative reflexive autoethnographic field notes, including daily three-page journal entries coded with weekly reflective summaries; work logs/mind maps with visual arts; a poetry chapbook fashioned from a gardening log; assignments from the massive/microscopic global autoethnographic work group; Facebook posts, messages, and texts; and new and existing biweekly-alternating and monthly programs broadcast by my New York City dance organization. As a kinesthetic learner; performing artist; professional dancer, choreographer, writer, and researcher; and not-for-profit organizational founder and leader, over these 5months I also shifted-up my screen-bound stasis through nonverbal-kinesthetic inquiry and reflective practices, recognizing my stress and to ground myself. My privileged self-care included a progressive seasonal menu 6 to 7 days a week: forest-bathing/hiking on a two-mile back ridge, taking an online classical ballet barre, walking a 4.8-mile road loop, and swimming open-water 90 mins in a glacial lake. While I think I know how to clean up well enough (critical when writing grant proposals and funding requests for government, foundation, corporate, and individual donors), my COVID-19 performative spoken-word rants and nonverbal-kinesthetic improvisational-movement compositions erupted suddenly, unexpectedly, messily with emotional feelings: fear, loss, distress, outrage, sadness, despair, and uplift. Personal and national histories of “healed and healing” lifelong and intergenerational physical and emotional traumas can surface unexpectedly. I see more clearly now through this performative reflexive autoethnographic process how *mine*, were laced with *our*, traumas of suppressed, rewritten, and tragically unacknowledged U.S., hemispheric, and global histories.	I witness the tail winds I witness the tail winds of Hurricane Isaias blowing northeast toward the Berkshires while swimming for 90 minutes in the glacial lake that has become my salvation, de facto lover, and source of feelings of transforming into a fish, illumination of my entire skeleton, my adult rebirth. (DeGarmo, 2020d) I start crying underwater I start crying underwater so the neighbors won’t become alarmed. I find myself involuntarily sobbing and wailing in the water. One night after swimming I suddenly need to pull to the side of the road and stop. My grief overwhelms me. I can’t see to drive and cry at the same time. (DeGarmo, 2020c)
